# Supplementary material for: Ecosystem natural capital accounting: The landscape approach at a territorial watershed scale
Source: Quant Plant Biol. 2022 Nov 2;3:e24. doi: 10.1017/qpb.2022.11 (PMC10095874; doi:10.1017/qpb.2022.11)
Supplement: Supplementary file 1 [file S263288282200011Xsup001.pdf]

Supplementary File S1. The Rhône river watershed, administrative – departments (left, blue lines represent department borders and the red lines represent the watershed**) and physical (right) maps.** The physical map shows the main Rhône affluents and main cities position along the rivers. The area of study covers 98,622 km^2, this area encompasses several valleys and rivers in three major regions in Europe, alpine, continental, and Mediterranean (Olivier et al., 2009). The watershed is shared by France and Switzerland, the Rhône river originates in the Swiss Alps and runs through south-eastern France towards the Mediterranean Sea. The total average annual precipitation in the north-south transect valley is approximately 600 mm yr^{-1}, the eastern areas and the mountains receive the highest annual precipitation amounts from 1400 to 2000 mm on average (Diodato et al., 2016).

Source for the physical map - https://webzine.one/boutique/cartes-ign/cartes-valleedurhone-ign/

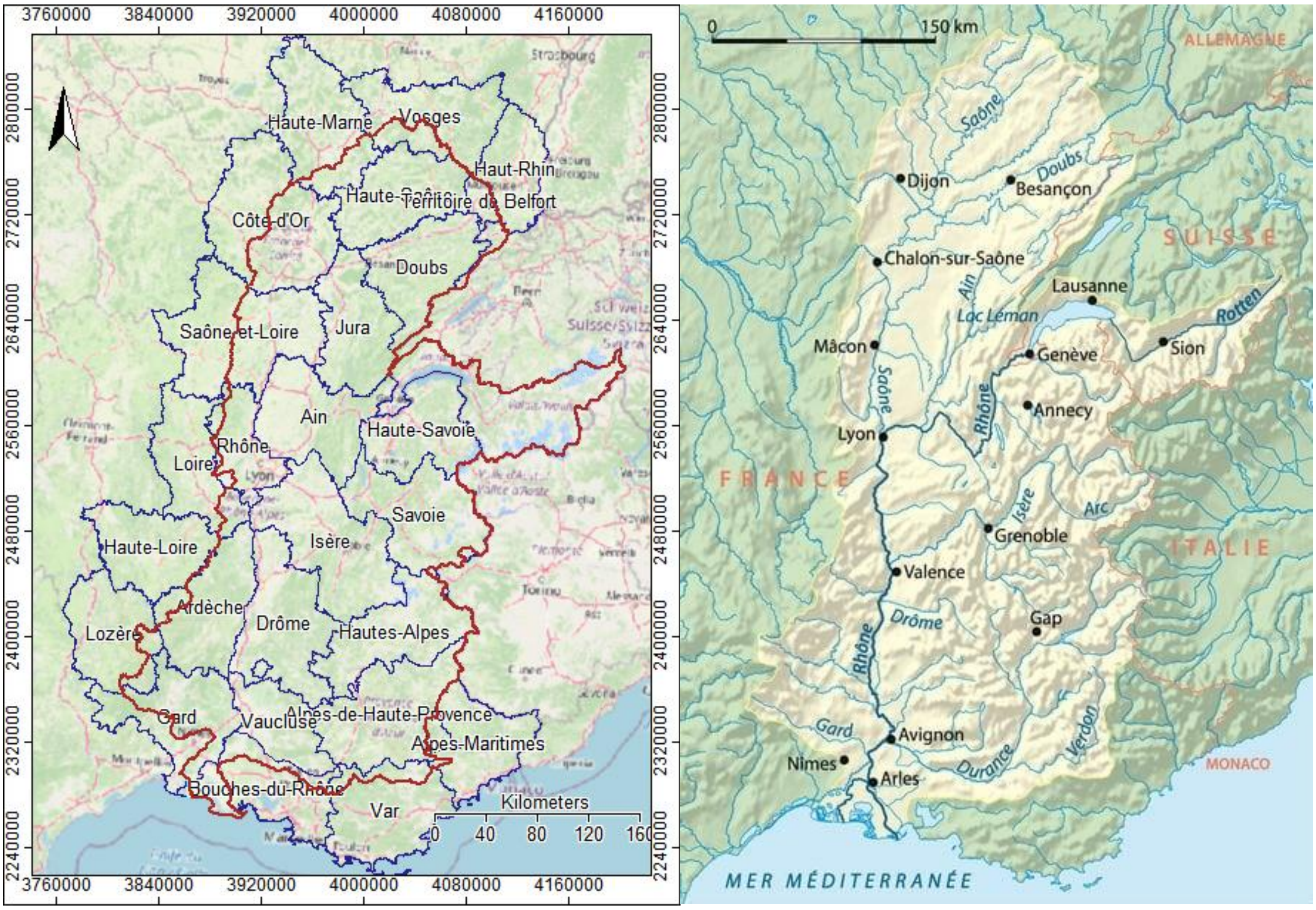

Supplementary File S2. Workflow of the bio-carbon accounting items, stocks, flows, and use with hyperlinks to data sources (a) and *Bio-carbon balance sheet 2000* as an example of accounting table (b). ENCA operates in conformity with "international statistical standards" (e.g., SEEA-EA chapters on the general framework and accounts in physical terms) adopted by the UN Statistical Commission (UN SEEA-EEA, 2021).

(a) Workflow to generate the annual carbon account with hyperlinks to data sources.

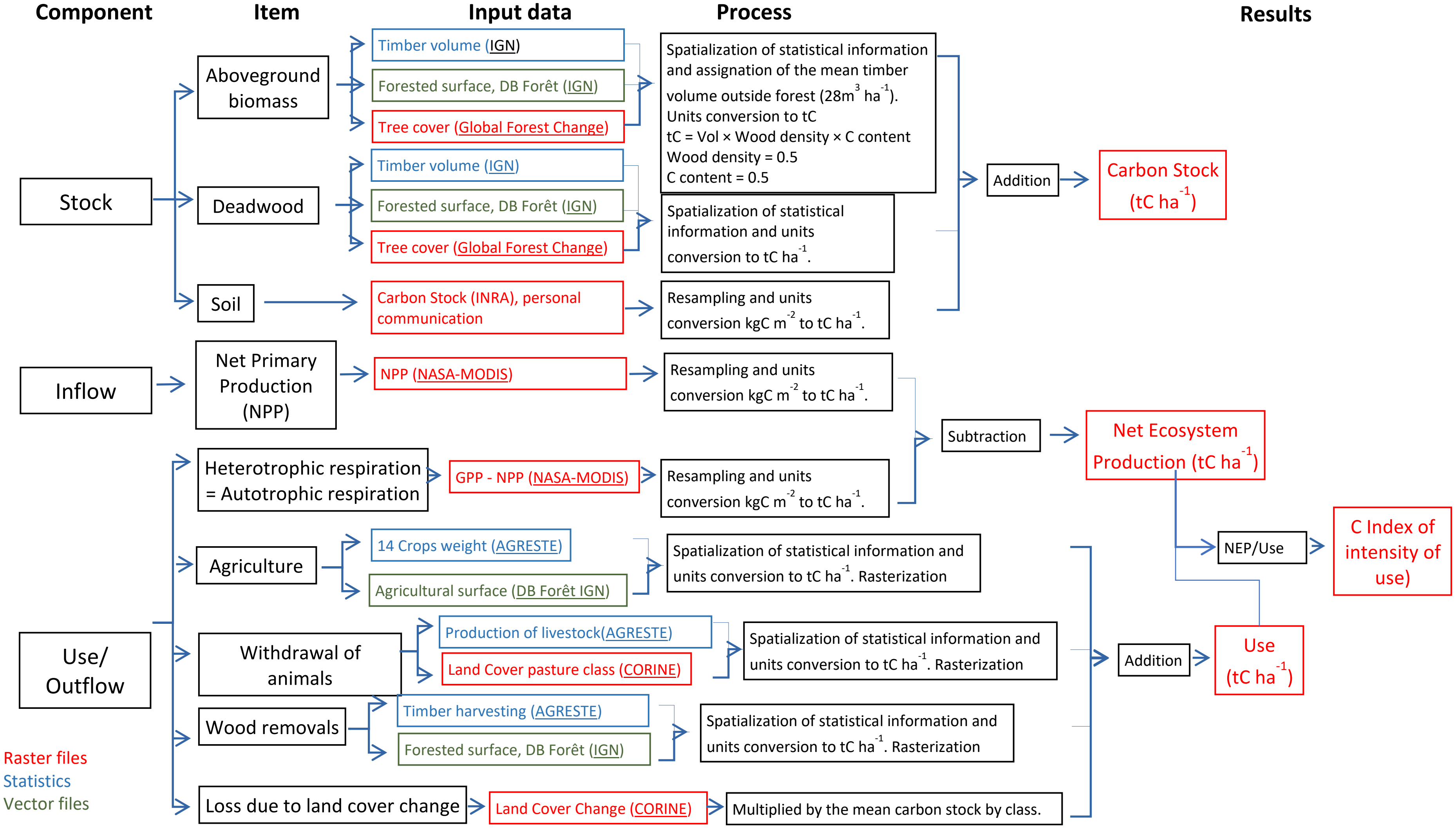

(b) Carbon balance sheet 2000

Ecosystem Carbon Account 2000												
	SEEA-EEA & ENCA-QSP land cover ecosystem units	DLCT 1	DLCT 2	DLCT 3	DLCT 4	DLCT 5	DLCT 6	DLCT 7	DLCT 8	DLCT 9	DLCT 10	***TOTAL tC***
		Artificial	Agricultural land	Pastures	Agricultural mosaics	Forest	Natural grassland	Shrubland and heathland	Open spaces	Glaciers and perpetual snow	Wetlands and water bodies	
	Area (ha)	136381	1049546	549219	968854	4474305	395391	543494	646937	55395	147190	
	IPCC land use classification	*SL = Settlements*	*CL = Cropland*			*GL = Grassland*	*FL = Forest Land*	*OL = Other Land*			*WL = Wetlands*	
**I. Ecosystem Carbon Basic Balance**												
C1	Opening Stocks	2394968,93	19306551,73	12708491,72	23082403,45	217906007,36	15866385,97	17336726,85	14514435,05	128467,41	2344446,80	325 588 885,26
C2.a	*NEP (Net Ecosystem Production) = C2.3-C2.4*	*49913,25*	*1874303,87*	*1190889,82*	*1868262,85*	*11403294,39*	*605947,82*	*1086632,00*	*532978,13*	*2871,95*	*132032,50*	*18 747 126,58*
C2	Total inflow of biocarbon (gains) = C2.a+C2.b	49913,25	1874303,87	1190889,82	1868262,85	11403294,39	605947,82	1086632,00	532978,13	2871,95	132032,50	18 747 126,58
C3.a	*Harvest of agriculture crops, wood & other vegetation*	*40119,66*	*1255708,70*	*406051,71*	*771510,78*	*2561660,07*	*40270,81*	*74251,70*	*17489,67*	*23,62*	*36672,87*	5 203 759,60
C3.b	*Withdrawals of secondary biocarbon*	*1560,06*	*19081,98*	*113466,95*	*39396,96*	*238509,35*	*2478,96*	*1697,58*	*534,24*	*0,00*	*1479,24*	
C3	Total withdrawals of biocarbon = C3.a+C3.b	41679,72	1274790,68	519518,67	810907,74	2800169,42	42749,77	75949,28	18023,91	23,62	38152,11	5 621 964,92
C4	Net indirect anthropogenic losses of biocarbon & biofuel com	86052,62	1798,03	0	0	0	0	0	0	0	0	
C5	*Total use of ecosystem biocarbon = C3+C4*	*127732,34*	*1276588,70*	*519518,67*	*810907,74*	*2800169,42*	*42749,77*	*75949,28*	*18023,91*	*23,62*	*38152,11*	*5 709 815,56*
C6	*Natural processes and disturbances*											
C7	Total outflow of biocarbon (losses)	127732,34	1276588,70	519518,67	810907,74	2800169,42	42749,77	75949,28	18023,91	23,62	38152,11	5 709 815,56
C8.1	*Net Ecosystem Carbon Balance 1, NECB 1 [Flows] = Inflows - Outflows = C2-C7*	*-77 819,09*	*597 715,17*	*671 371,15*	*1 057 355,12*	*8 603 124,97*	*563 198,05*	*1 010 682,72*	*514 954,22*	*2 848,33*	*93 880,39*	*13 037 311,02*
C8.2	Adjustment and reappraisals											
C8.3	*NECB 2 [Stocks] = Change of biocarbon stocks*											
C9	Closing Stocks = C1+C8.1+C8.2 or = C1+C8.3	2317149,84	19904266,90	13379862,87	24139758,56	226509132,32	16429584,02	18347409,56	15029389,27	131315,74	2438327,19	338 626 196,28
**II. Accessible Resource Surplus**												
C2	*Total inflow of biocarbon (gains) = C2.a+C2.b*	49913,25	1874303,87	1190889,82	1868262,85	11403294,39	605947,82	1086632,00	532978,13	2871,95	132032,50	18 747 126,58
C10	*Accessibility net correction*											
C11	Net Ecosystem Accessible Carbon Surplus = C2 + C10	49913,25	1874303,87	1190889,82	1868262,85	11403294,39	605947,82	1086632,00	532978,13	2871,95	132032,50	18 747 126,58
**III. Total Uses of Ecosystem Bio and Geo-Carbon**												
C5	*Total use of ecosystem biocarbon = C3+C4*	*127732,34*	*1276588,70*	*519518,67*	*810907,74*	*2800169,42*	*42749,77*	*75949,28*	*18023,91*	*23,62*	*38152,11*	*5 709 815,56*
**IV. Table of indexes of intensity of use and ecosystem health**												
SCU	Intensity of carbon use = C11/C5	0,39	1,47	2,29	2,30	4,07	14,17	14,31	29,57	121,57	3,46	

Supplementary File S3. Software packages, data structure, and geomatic treatment information.

Assimilation and data integration of statistics and geodata. Available data were heterogeneous (e.g., acquisition date, coverage, consistency and content, with a wide variety of data resolutions). 1ha (100m x 100m) has been selected as basic spatial unit or resolution. All geographical input data were systematically resampled to pixels of 100m x 100m.

The panels focus on the data formats employed with ENCA, the tools to integrate the accounts, and the production of grids for data analysis. Concerning data management, the fast development of big data is a game changer in this domain raising challenges in terms of consistency, exhaustiveness and stability over time, and constraints in terms of accessibility of commercial data (an issue, for example, with data on water supply and management). Big data sets compiled for commercial purposes can generate bias in data accessible through intermediation platforms. In parallel, information technology is supporting the development of open access projects controlled by citizens, such as the OpenStreetMap (used in this work) or the Global Biodiversity Information Facility (https://www.gbif.org/fr/).

Software Packages employed during this work.

The ENCA-QSP tests have been carried out using the following software packages according to the best convenience of use for specific tasks:

QGIS and SAGA-GIS for geographical information processing. These two packages are partly integrated and their main data formats are fully compatible. They are .shp (the ESRI shapefile format), .tiff and .sdat (the SAGA grid format, read by QGIS). These are two powerful freeware packages fit for the purpose of accounting. QGIS is particularly fit for cartographic work with a powerful shape files editor. SAGA targets scientific calculation needs, with a library of circa 500 modules. De facto, ENCA can be implemented with other GIS packages, e.g. with ArcGis

SAGA-GIS: System for Automated Geoscientific Analyses
http://www.saga-gis.org/en/index.html

Ref.: Conrad, O., Bechtel, B., Bock, M., Dietrich, H., Fischer, E., Gerlitz, L., Wehberg, J., Wichmann, V., and Böhner, J. (2015): System for Automated Geoscientific Analyses (SAGA) v. 2.1.4, Geosci. Model Dev., 8, 1991-2007, doi:10.5194/gmd-8-1991-2015.

QGIS : https://qgis.org/en/site/

MS Excel and LibreOffice Calc have been used for integrating accounts. Their formats are fully compatible. Final integration and data management has been carried out with PostgreSQL and PostGIS.

PostGIS for geographic objects to the PostgreSQL object-relational database : https://postgis.net/

Schematics of the production of grids with same 1-ha pixel-size for data analysis

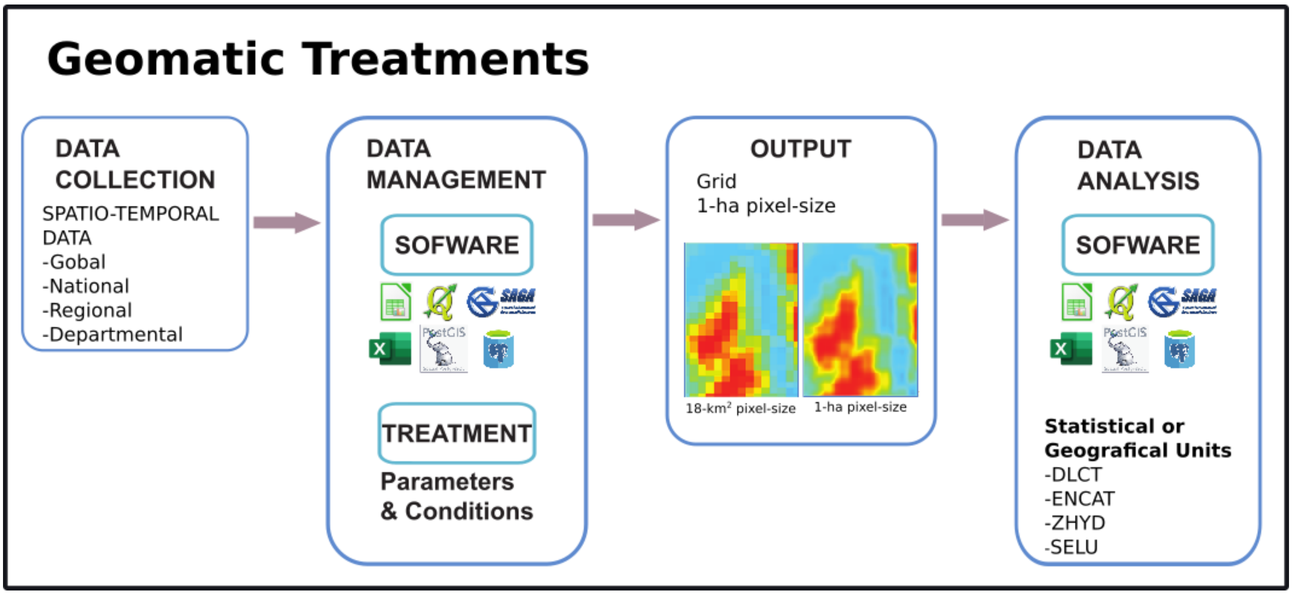
Geomatic Treatments
DATA COLLECTION
SPATIO-TEMPORAL DATA
-Gobal
-National
-Regional
-Departmental
DATA MANAGEMENT
SOFWARE
TREATMENT
Parameters & Conditions
OUTPUT
Grid
1-ha pixel-size
18-km² pixel-size
1-ha pixel-size
DATA ANALYSIS
SOFWARE
Statistical or Geografical Units
-DLCT
-ENCAT
-ZHYD
-SELU

Supplementary File S4. Water and river accounts. (a) the intersection of Dominant Land Cover Types (DLCT, coloured areas) and river basin boundaries (ENCAT, white lines) for producing SELUs, the statistical-geographic unit that integrates land and river entities. **(b)** Detailed structure of ENCA Water Account (Weber, 2014).

(a) Intersection of Dominant Land Cover Types (DLCT, coloured areas) and ENCAT (white lines) for producing SELUs.

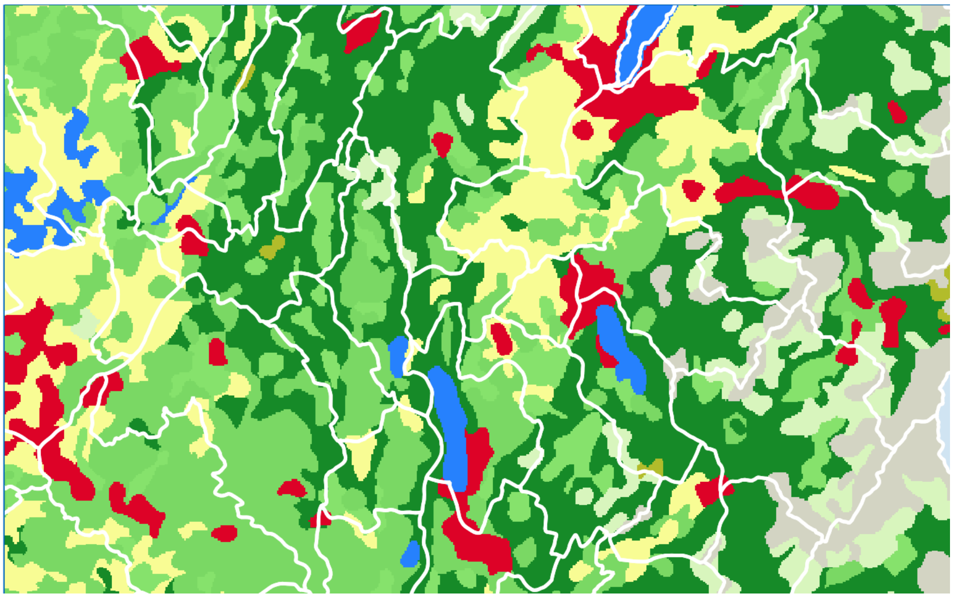

(b) Structure of ENCA Water Account.

Accounting category	Variables and intermediate indices	Synthetic indices
I. Ecosystem Water Basic Balance	Stocks<br>Primary and secondary production of water<br>Transfers between water bodies and basins<br>Actual Evapotranspiration<br>Abstraction of water, supply and use<br>Returns to waste water and losses	*Total Inflow of water*<br>*Net Ecosystem Water Balance*
II. Accessible Resource Surplus	Total renewable water resources<br>Accessible stock carried over<br>Restrictions of use<br>Other accessibility corrections	*Net Accessible Water Resource*
III. Total uses of water	Total use of ecosystem water: blues, grey & green water<br>Imports/water commodities contents<br>Imports/ embedded water	*Total use of ecosystem water*<br>*Direct use Of water*<br>*Total water requirement*
IV. Table of Indices of Intensity of use and Ecosystem Health	Intensity of water use<br>Composite ecosystem water health Index	*Water Internal ecological unit value*

Supplementary File S5. Ecosystem infrastructure accounts.

(a) Structure of ENCA Ecosystem Infrastructure Functional Services Accounts. The detailed description of each accounting category (accounting tables I to IV) for land and river ecosystems and services is given in section (c) below.

(b) ***Land and River system indicators (NLEP*** and ***NREP):*** comparative outlook and subsequent integration to generate the Total Ecosystem Infrastructure Potential (TEIP). The ***index of greenness*** is defined by scoring Land Cover classes from CORINE according to their artificiality/naturalness. The ***index of landscape connectivity / fragmentation*** is the effective mesh size **(**m_{eff}; Mosser et al., (2007), reflecting the probability that two points chosen randomly in a region are connected. The SELUs are clipped with fragmentation elements (motorways and railways) according to Open Street Map. The ***index of natural conservation value (NATURILIS)*** is computed as the sum of datasets on protected areas from the *Inventaire National du Patrimoine Naturel*, assigning them a score according to their protection status (Weber et al., 2008). The ***Net River Ecosystem Potential (NREP)*** combines the River Condition Potential and the index of natural conservation value for rivers (NATRIV) through the geometric mean. The ***Rivers Condition Potential*** is a combination of rivers length and discharge weighted by the ecological status index (EEA, 2018) of Hydrological Units (UZHYD). To calculate ***NATRIV,*** rivers from BD Carthage are intersected with NATURILIS. The ***Total Ecosystem Infrastructure Potential (TEIP)*** is the aggregation (sum) of NLEP and NREP by SELU. Thus, TEIP integrates changes in NLEP, related to terrestrial attributes (greenness, areas of conservation, and fragmentation) with changes in NREP, related to river attributes (river condition potential and areas of conservation for rivers) (Weber, 2014).

(**c)** The ***Total Ecosystem Infrastructure Potential (TEIP).*** Codes on the left of panels correspond to accounting table nomenclature. There are four steps of integrating NLEP and NREP in the synthetic indicator TEIP: a basic balance sheet (I), a balance sheet with the main variables / intermediate indices used to calculate the Accessible Ecosystem Infrastructure Potential (II), an accounting sheet with the variables that determine the overall access to ecosystem infrastructure services (III), and a table assembling the indices of ecosystem infrastructure intensity of use and ecosystem health (IV). Ecosystem Health indices complement measurements of the ecosystem infrastructure condition with information on changes in species diversity.

Detailed guidelines are described in Weber (2014) and the European Habitats Directive (2012).

(a) Detailed structure of ENCA Ecosystem Infrastructure Functional Services Account.

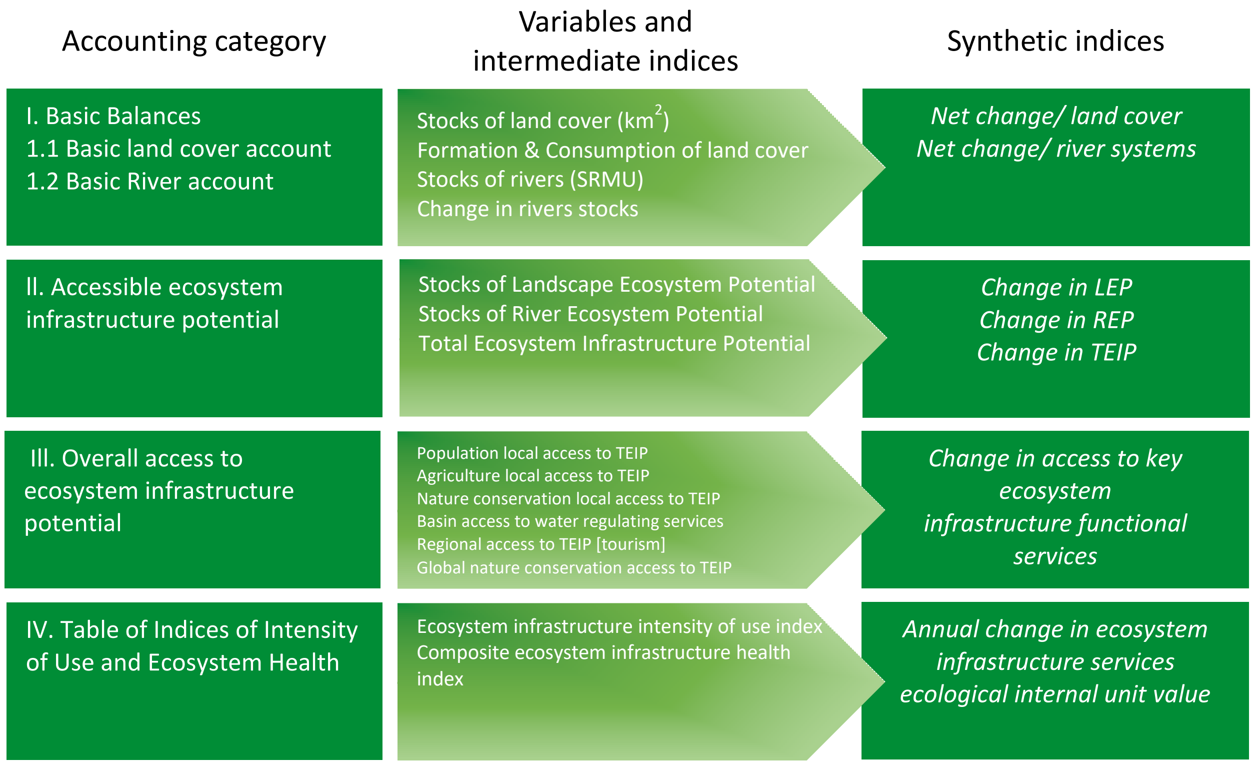

(b) Land and River system indicators: comparative outlook and integration to generate the Total Ecosystem Infrastructure Potential (TEIP).

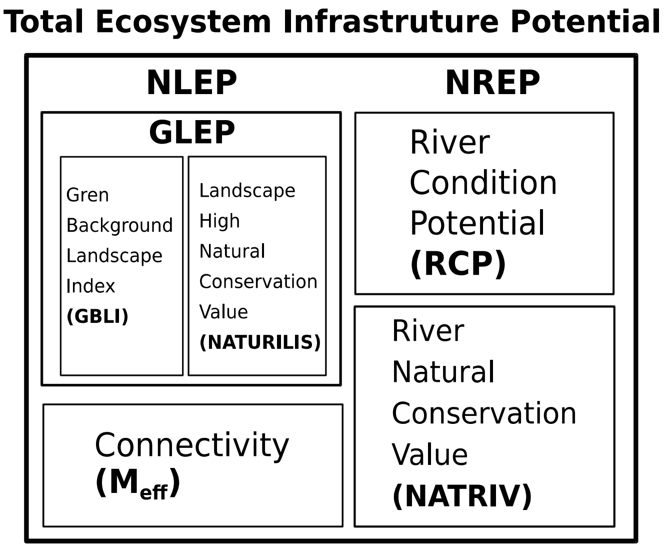

(c)

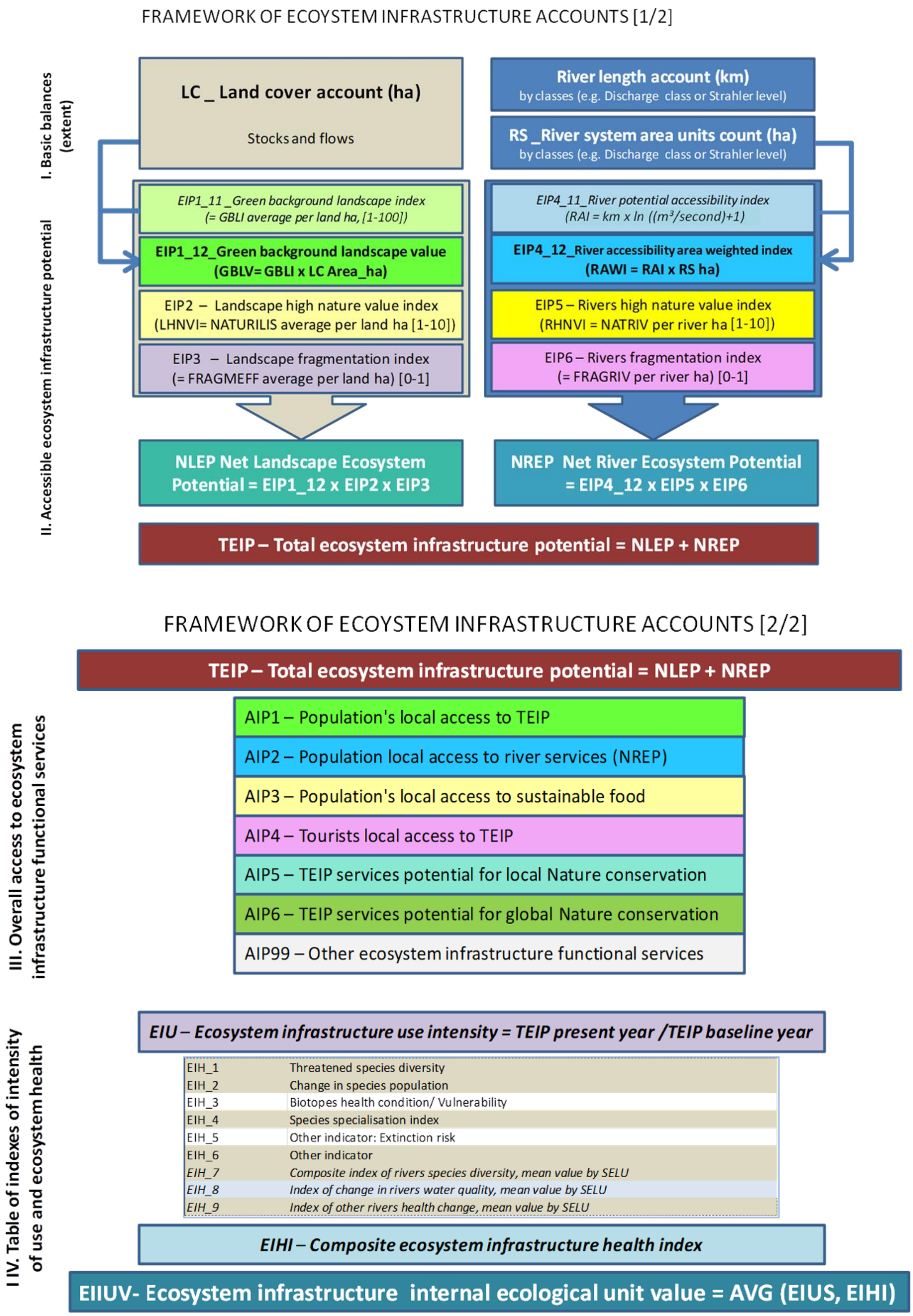
FRAMEWORK OF ECOYSTEM INFRASTRUCTURE ACCOUNTS [1/2]
I. Basic balances (extent)
II. Accessible ecosystem infrastructure potential
LC _ Land cover account (ha)
Stocks and flows
River length account (km)
by classes (e.g. Discharge class or Strahler level)
RS _River system area units count (ha)
by classes (e.g. Discharge class or Strahler level)
EIP1_11 _Green background landscape index
(= GBLI average per land ha, [1-100])
EIP1_12_Green background landscape value
(GBLV= GBLI x LC Area_ha)
EIP2 – Landscape high nature value index
(LHNVI= NATURILIS average per land ha [1-10])
EIP3 – Landscape fragmentation index
(= FRAGMEFF average per land ha) [0-1]
EIP4_11_River potential accessibility index
(RAI = km x ln ((m³/second)+1)
EIP4_12_River accessibility area weighted index
(RAWI = RAI x RS ha)
EIP5 – Rivers high nature value index
(RHNVI = NATRIV per river ha [1-10])
EIP6 – Rivers fragmentation index
(= FRAGRIV per river ha) [0-1]
NLEP Net Landscape Ecosystem Potential = EIP1_12 x EIP2 x EIP3
NREP Net River Ecosystem Potential = EIP4_12 x EIP5 x EIP6
TEIP – Total ecosystem infrastructure potential = NLEP + NREP
FRAMEWORK OF ECOYSTEM INFRASTRUCTURE ACCOUNTS [2/2]
TEIP – Total ecosystem infrastructure potential = NLEP + NREP
III. Overall access to ecosystem infrastructure functional services
AIP1 – Population's local access to TEIP
AIP2 – Population local access to river services (NREP)
AIP3 – Population's local access to sustainable food
AIP4 – Tourists local access to TEIP
AIP5 – TEIP services potential for local Nature conservation
AIP6 – TEIP services potential for global Nature conservation
AIP99 – Other ecosystem infrastructure functional services
I IV. Table of indexes of intensity of use and ecosystem health
EIU – Ecosystem infrastructure use intensity = TEIP present year /TEIP baseline year
EIH_1 Threatened species diversity
EIH_2 Change in species population
EIH_3 Biotopes health condition/ Vulnerability
EIH_4 Species specialisation index
EIH_5 Other indicator: Extinction risk
EIH_6 Other indicator
EIH_7 Composite index of rivers species diversity, mean value by SELU
EIH_8 Index of change in rivers water quality, mean value by SELU
EIH_9 Index of other rivers health change, mean value by SELU
EIHI – Composite ecosystem infrastructure health index
EIIUV- Ecosystem infrastructure internal ecological unit value = AVG (EIUS, EIHI)

Supplementary File S6. Lessons from biodiversity, river status analyses, and bio-carbon magnitudes. The ensemble can be considered with respect to the outlook of land-use patterns, agriculture in particular, in Figure 1c.

Comparative representations of Biodiversity evaluation (a-d). In all cases the state and pressures on ecosystems are used to evaluate biodiversity (Index of Ecosystem Health, *with data from* Article 17, European Habitats Directive, 2012). **(a)** ***Species biodiversity trend index for 2006*** (this work)**.** Index values larger than 1 (green and grey) reflect that positive expert's diagnoses on species biodiversity outdo negative ones, while values smaller than 1 (yellow to red) show degradation, which is compared with **(b)** The ***gradients of pressures on biodiversity***, namely soil artificialization, resource overexploitation, climate change, pollution, and invasive alien species**,** according to *Observatoire National de la Biodiversité*, OBN (2017). **(c)** One pressure indicator, the ***Fragmentation (Mesh effective size) of natural areas,*** shows the fragmentation by departmental forestry region (OBN, 2017) which is compared with **(d)** The ***Mesh effective size of the Rhône*** watershed by SELU (this work) - mesh size of 330.62 km^2 per SELU.

(**e**) ***Change in the Ecological status of all rivers*** by hydrological units ENCAT. The index assembles chemical, biological, and functional parameters. Warm values indicate degradation.

(**f**) Evolution of the ENCA ***River Condition Potential*** (%, over the 2009-2015 period), according to river classes. The degradation of the potential occurred irrespective of river class.

(g) ***Bio-carbon accounts with comparative absolute values for stocks, flows, and use***. Trees are the main carbon stock, with 20% of GPP entering the flow as NEP, and the use corresponding to approx. 33% of the NEP. Of note, the general stocks represent a relatively consistent buffer in terms of GPP/NPP levels to sustain the production of the ecosystem, despite unsustainable use due to pressures more readily and specifically affecting agriculture and forest sourced biomass. Abbreviations: GPP - Gross Primary Production; NEP – Net Ecosystem Production; NPP - Net Primary Production.

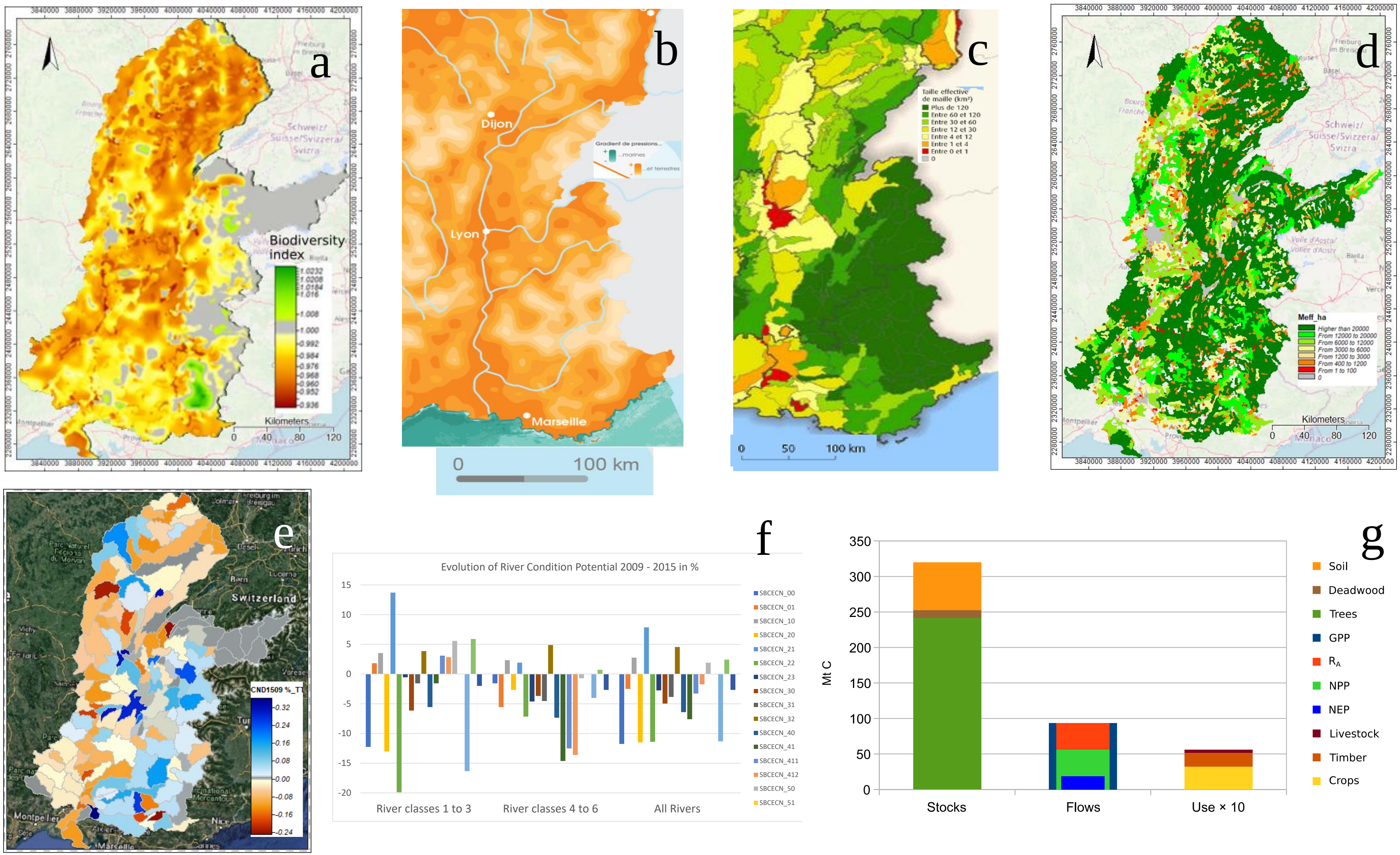
a
Biodiversity index
Kilometers
0 40 80 120
b
Dijon
Lyon
Marseille
0 100 km
c
Taille effective de maille (km²)
Plus de 120
Entre 60 et 120
Entre 30 et 60
Entre 12 et 30
Entre 4 et 12
Entre 1 et 4
Entre 0 et 1
0
0 50 100 km
d
Meff_ha
Higher than 20000
From 12000 to 20000
From 6000 to 12000
From 3000 to 6000
From 1200 to 3000
From 400 to 1200
From 1 to 100
0
Kilometers
0 40 80 120
e
Switzerland
CND1509 %_TT
f
Evolution of River Condition Potential 2009 - 2015 in %
River classes 1 to 3
River classes 4 to 6
All Rivers
g
Mt C
Stocks
Flows
Use × 10
Soil
Deadwood
Trees
GPP
R_A
NPP
NEP
Livestock
Timber
Crops
